# Clinical outcomes of relapsed and refractory Hodgkin lymphoma patients after contemporary first-line treatment: a German Hodgkin Study Group analysis

**DOI:** 10.1038/s41375-021-01442-8

**Published:** 2021-10-09

**Authors:** Paul J. Bröckelmann, Horst Müller, Sarah Gillessen, Xiaoqin Yang, Larissa Koeppel, Veronika Pilz, Patricia Marinello, Peter Kaskel, Monika Raut, Michael Fuchs, Peter Borchmann, Andreas Engert, Bastian von Tresckow

**Affiliations:** 1 Department I of Internal Medicine, Center for Integrated Oncology Aachen Bonn Cologne Duesseldorf (CIO ABCD), University of Cologne, Cologne, Germany; 2 German Hodgkin Study Group (GHSG), University of Cologne, Cologne, Germany; 3 Cancer Center Cologne Essen (CCCE), Cologne/Essen, Germany; 4 Merck & Co., Inc, Kenilworth, NJ USA; 5 MSD Sharp & Dohme GmbH, Haar, Germany; 6 Department of Hematology and Stem Cell Transplantation, West German Cancer Center, University Hospital Essen, University of Duisburg-Essen, Essen, Germany

**Keywords:** Chemotherapy, Hodgkin lymphoma

## Abstract

To evaluate patterns of rrHL after contemporary first-line treatment we studied 409 patients with first rrHL (HD13: *n* = 87, HD14: *n* = 118, HD15: *n* = 188, HDR3i: *n* = 51) at a median age of 37.4 years (18.4–76.8) from the GHSG database. Time to first relapse was ≤12 months in 49% and stage III/IV rrHL present in 52% of patients. In total, 291 patients received high-dose chemotherapy and autologous stem-cell transplantation (ASCT) and intended ASCT failed in 38 patients. ASCT was primarily not intended in 80 patients largely due to low risk disease or age/comorbidities. Overall, 10-year progression-free (PFS) and overall survival (OS) rates after first relapse were 48.2% (95% CI 41.9–54.2%) and 59.4% (95% CI 53.0–65.2%), respectively, with significant differences between subgroups. Inferior survival was observed with no ASCT due to advanced age/comorbidities (five-year PFS 36.2%, 95% CI 17.7–55.0%) or failure of salvage therapy (five-year PFS 36.3%, 95% CI 19.7–53.2%). Similarly, presence of primary refractory disease or stage IV at rrHL conferred inferior survival. In patients with low-risk disease, however, survival appeared favorable even without ASCT (10 y PFS 72.6%, 95% CI 53.7–84.8%). We herein confirm the curative potential of current rrHL treatments providing a robust benchmark to evaluate novel therapeutic strategies in rrHL. Approximately 50% of rrHL patients experienced a consecutive relapse.

## Introduction

Classical Hodgkin lymphoma (HL) is highly curable when adequately treated with risk-adapted contemporary 1st-line therapy. Depending on disease extent and clinical risk factors, patients are usually assigned to the early-stage favorable, unfavorable or advanced-stage HL risk groups [1]. With doxorubicin, bleomycin, vinblastine and dacarbazine (ABVD) and/or bleomycin, etoposide, doxorubicin, cyclophosphamide, vincristine, procarbazine and prednisolone (BEACOPP) as chemotherapeutic backbones of HL 1st-line therapy, long-term progression-free (PFS) and overall survival (OS) rates exceeding 90% are observed [2–4]. Outcomes however are less favorable in older patients or those ineligible for intensified approaches and treatment of relapsed or refractory HL (rrHL) remains a significant clinical challenge.

Salvage chemotherapy with dexamethasone, cytarabine and cisplatin (DHAP) or ifosfamide, carboplatin and etoposide (ICE) followed by high-dose chemotherapy and autologous stem-cell transplantation (ASCT) are standard of care (SOC) in eligible rrHL patients [5–7]. Other conventional treatment options include polychemotherapy without consolidative ASCT [8], other mostly palliative chemotherapies [9–11], allogeneic SCT (alloSCT) [12] or radiotherapy (RT) for localized relapse [13]. Numerous clinical risk factors (RF) have been identified for inferior PFS and OS in rrHL [14]. These RF usually reflect adverse HL characteristics (e.g. stage III/IV at relapse, bulky disease, extranodal involvement), patient features (e.g. male sex, advanced age, ECOG performance status) or chemosensitivity of disease (e.g., time to relapse (TTR), number of salvage therapies, response to salvage therapy) and may be combined in prognostic indices [15]. The prognosis of rrHL in older or ASCT-ineligible as well as multiply relapsed patients has historically been unsatisfactory, especially in high-risk patients e.g., with TTR < 3 months after end of 1st-line treatment [11, 16, 17].

During the last decade, the anti-CD30 antibody-drug-conjugate brentuximab vedotin (BV) [18] and more recently the anti-PD1 antibodies nivolumab [19] and pembrolizumab [20] have been approved for rrHL based on a favorable safety profile and high efficacy. Approval was based on non-controlled phase II trials and due to the relative paucity of rrHL patients and heterogenous SOC, direct comparisons in randomized trials are challenging. The randomized international AETHERA and KEYNOTE-204 trials leading to approval of BV as consolidative treatment in high-risk patients after ASCT [21] and showing superiority of pembrolizumab over BV [22], respectively, are recent welcome exceptions. Despite these two well controlled clinical trials, the potential benefit of the majority of current and novel targeted agents in rrHL compared to SOC will only be measurable indirectly and relative to outcomes of patient cohorts treated in routine care [23]. This challenge especially applies to specific groups of rrHL or clinical situations such as older and/or ASCT-ineligible or multiply relapsed patients. The currently available data of first rrHL is limited due to either outdated preceding 1st-line treatments, small sample size, monocentric cohorts or focus only on patients successfully undergoing ASCT. There hence is a lack of large-scale contemporary data on patient, disease and treatment characteristics as well as associated outcomes.

The present analysis aims to provide a comprehensive evaluation of disease characteristics, treatment patterns and clinical outcomes of first rrHL after contemporary 1st-line treatment in a multicenter setting. It thereby informs decision making in routine care, patient counseling and and may serve as a robust benchmark to evaluate the relative benefit of novel therapies or strategies.

## Methods

Patients with first episode of rrHL documented and treated in routine care at the local physicians discretion during follow-up after standard of care 1st-line treatment in the randomized phase III GHSG HD13 (ISRCTN registry: ISRCTN63474366) [24], HD14 (ISRCTN04761296) [25] or HD15 (ISRCTN32443041) [26] trials were identified in the GHSG database. Briefly, early-stage favorable patients received 2x ABVD, 2x AVD, 2x ABV or 2x AV each followed by 30 Gy involved-field radiotherapy (IF-RT) in HD13 and the trial failed to show non-inferior PFS of the ABVD variants [24]. Of note, patients randomized to the ABV and AV groups were excluded from the present analysis due to 1^st^-line treatment with non-SOC chemotherapy. Early-stage unfavorable patients were treated with either 4x ABVD or 2x BEACOPPesc + 2x ABVD (“2 + 2”) each followed by 30 Gy IF-RT and superior five-year PFS was observed with 2 + 2 in HD14 [25]. In HD15, advanced-stage patients experienced superior disease control and OS after 6x BEACOPPesc vs. 8x BEACOPPesc and IF-RT was applied to positron emission tomography (PET)-positive residual tissue in both groups. Relapsed or refractory HL patients treated in the recent GHSG HDR3i phase II trial (Clinical Trials: NCT01453504), failing to show superior complete remission rates with everolimus + DHAP compared to DHAP alone prior to planned ASCT [27], were additionally included. All patients had provided written informed consent at enrollment into the respective clinical trials, which were approved by the responsible ethics committees and conducted in accordance with good clinical practice (GCP) requirements.

Patient, disease and treatment characteristics were obtained from the trial database and pseudonymized patient files where available. Patients with insufficient data on rrHL or no follow-up after rrHL diagnosis were excluded from the analysis. Response to rrHL treatment was assessed locally based on the imaging modality and response criteria available at the time of assessment. OS was measured from date of rrHL to date of death for any reason and PFS was calculated from date of rrHL to date of progressive HL, relapse of HL or death for any reason, whichever occurred first, with both endpoints censored at last available follow-up. Predefined subgroup analyses were performed in patients intended to undergo ASCT with or without consolidative treatment or not intended to receive ASCT. Descriptive statistics and Kaplan–Meier estimates were used to describe the different cohorts and all GHSG authors had access to the primary data. All statistical analyses were performed with SAS 9.4 and if present, missing data are reported.

## Results

**Fig. 1:**
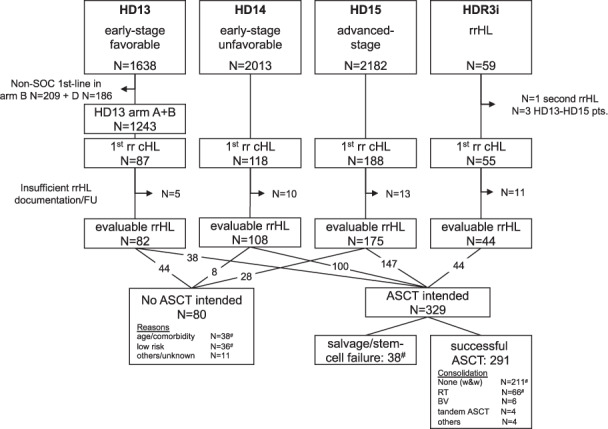
Patient flow chart of study population and treatment for rrHL. ^#^rrHL patient subgroups selected for detailed analysis. Abbreviations: SOC standard of care, rrHL relapsed or refractory Hodgkin lymphoma, cHL classical Hodgkin lymphoma, FU follow-up, ASCT high-dose chemotherapy and autologous stem-cell transplantation, w&w: watch and wait, RT radiotherapy, BV brentuximab vedotin.

Among 5,777 HL patients in the GHSG first-line trials HD13 to HD15 and 59 rrHL patients treated in the HDR3i trial, 409 patients were evaluable for first rrHL ocuring between May 2003 and March 2018. The largest group of rrHL patients was initially treated for advanced-stage HL in the HD15 trial (*n* = 175, 43%), followed by early-stage unfavorable disease treated in the HD14 trial (*n* = 108, 26%) and early-stage favorable patients enrolled in the HD13 trial (*n* = 82, 20%, Fig. 1). The median age at first rrHL of the predominantly male study population (*n* = 262, 64%) was 37.4 years (range 18–77 years). Time to relapse after end of first-line treatment was mostly >12 months (*n* = 208, 51%) and the majority of patients presented with advanced-stage III/IV at relapse. Roughly one third of patients had extranodal disease or B-symptoms at relapse (Table 1). A considerable number of patients were not considered for ASCT (*n* = 80, 20%), while the majority was intended to receive ASCT (*n* = 329, 80%) at the time of first rrHL (Fig. 1). Supplementary Table 1 summarizes the patient characteristics of the following five subgroups of interest: Patients not intended to undergo ASCT either due to age/comorbidities or low risk, patients intended to undergo ASCT but failing due to insufficient response to salvage treatment or stem-cell mobilization and patients successfully undergoing ASCT and either receiving consolidative RT or nor consolidation. The vast majority of patients intended to undergo ASCT received DHAP-based 2nd-line treatment with <5% each receiving ICE, Dexa-BEAM or other regimens. Supplementary Table 2 provides a detailed summary of the treatments applied in patients not intended to undergo ASCT at first rrHL.

**Table 1:** Patient and disease characteristics at first rrHL.

	No ASCT intended (*N* = 80)	ASCT intended (*N* = 329)	Total (*N* = 409)
Sex			
Female	33 (41%)	114 (35%)	147 (36%)
Male	47 (59%)	215 (65%)	262 (64%)
Age at rrHL			
Mean (SD)	48.1 (16.4)	36.3 (11.8)	38.6 (13.7)
Median	49.8	34.8	37.4
Range	(18.9-76.8)	(18.4-66.3)	(18.4-76.8)
Age > 60 years at rrHL			
No	58 (73%)	323 (98%)	381 (93%)
Yes	22 (28%)	6 (2%)	28 (7%)
Clinical stage at 1st diagnosis			
missing	0 (0%)	3 (1%)	3 (1%)
I	16 (20%)	10 (3%)	26 (6%)
II	36 (45%)	167 (51%)	203 (50%)
III	16 (20%)	65 (20%)	81 (20%)
IV	12 (15%)	84 (26%)	96 (23%)
B-symptoms at 1st diagnosis			
Missing	0 (0%)	4 (1%)	4 (1%)
No	61 (76%)	153 (47%)	214 (52%)
Yes	19 (24%)	172 (52%)	191 (47%)
Time to rrHL			
Missing	1	2	3
≤3 months	6 (8%)	68 (21%)	74 (18%)
3–12 months	20 (25%)	104 (32%)	124 (31%)
>12 months	53 (67%)	155 (47%)	208 (51%)
Clinical stage at rrHL			
Missing	0	3	3
I	25 (31%)	43 (13%)	68 (17%)
II	26 (33%)	101 (31%)	127 (31%)
III	15 (19%)	75 (23%)	90 (22%)
IV	14 (18%)	107 (33%)	121 (30%)
B-Symptoms at rrHL			
Missing	34	100	134
No	27 (59%)	159 (69%)	186 (68%)
Yes	19 (41%)	70 (31%)	89 (32%)
Bulk ≥ 5 cm at rrHL			
Missing	38	153	191
No	26 (62%)	103 (59%)	129 (59%)
Yes	16 (38%)	73 (41%)	89 (41%)
EN-disease at rrHL			
Missing	32	114	146
No	34 (71%)	153 (71%)	187 (71%)
Yes	14 (29%)	62 (29%)	76 (29%)
ECOG at rrHL			
Missing	50	149	199
0	24 (80%)	138 (77%)	162 (77%)
1	1 (3%)	24 (13%)	25 (12%)
2	5 (17%)	17 (9%)	22 (10%)
3	0 (0%)	1 (1%)	1 (0%)

*ASCT* high-dose chemotherapy and autologous stem-cell transplantation, *rrHL* relapsed or refractory classical Hodgkin lymphoma, *EN* extranodal disease, *SD* standard deviation.

Ultimately, a total of 111 patients did not undergo ASCT at first rrHL for reasons summarized in Supplementary Table 3. With multiple reasons documentable per patient, these were predominantly high age (in *n* = 24 patients, 22%) or comorbidities (*n* = 10, 9%) or low risk disease e.g., due to late relapse (*n* = 10, 9%) or localized rrHL treated with RT only (*n* = 21, 19%) among patients not considered for ASCT. In patients initially considered for ASCT main reason for ultimately not successfully undergoing ASCT at 1st rrHL was failure of salvage therapy (*n* = 30, 27%) or stem-cell mobilization (*n* = 3, 3%).

Locally assessed response rates to 2nd-line treatment are summarized in Table 2. The overall response rate (ORR) defined as the proportion of patients achieving a complete (CR) or partial response (PR) in the total study population was 82% with 56% and 26% of patients achieving a CR and PR, respectively. The highest CR rates were documented for patients not receiving ASCT due to low-risk disease (74%) or patients undergoing ASCT without consolidation (72%). CR rates were low in patients receiving ASCT and consolidative RT (23%) or experiencing salvage/stem-cell mobilization failure (14%), with progressive disease (PD) predominantly observed in the latter subgroup (48%).

**Table 2:** Investigator-assessed response rates to 2nd-line HL treatment.

	Low risk (*N* = 36)	High age/comorbidity (*N* = 33)	Salvage/SC-failure (*N* = 38)	ASCT with watch&wait (*N* = 211)	ASCT with RT consolidation (*N* = 66)	Total (*N* = 384)
Response						
Missing	1	5	9	25	9	49
CR	26 (74%)	12 (43%)	4 (14%)	133 (72%)	13 (23%)	188 (56%)
PR	6 (17%)	9 (32%)	4 (14%)	32 (17%)	36 (63%)	87 (26%)
SD	0 (0%)	2 (7%)	7 (24%)	3 (2%)	4 (7%)	16 (5%)
PD	3 (9%)	5 (18%)	14 (48%)	18 (10%)	4 (7%)	44 (13%)

*SC* stem cell harvest, *ASCT* high-dose chemotherapy and autologous stem-cell transplantation, *RT* radiotherapy, *CR* complete remission, *PR* partial remission, *SD* stable disease, *PD* progressive disease.

**Fig. 2:**
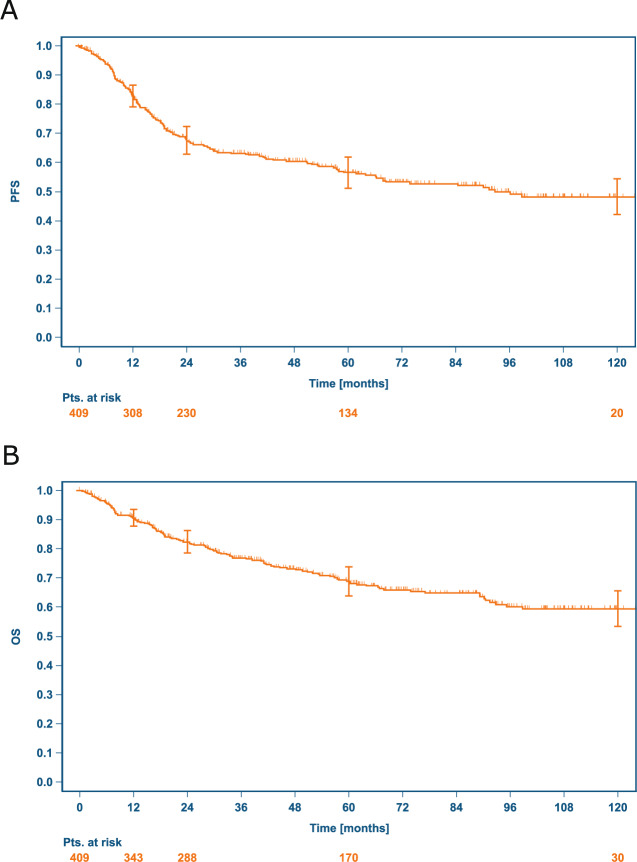
Kaplan–Meier PFS and OS curves after first rrHL. **A** Progression-free survival (PFS), **B** overall survival (OS), both from first rrHL.

**Fig. 3:**
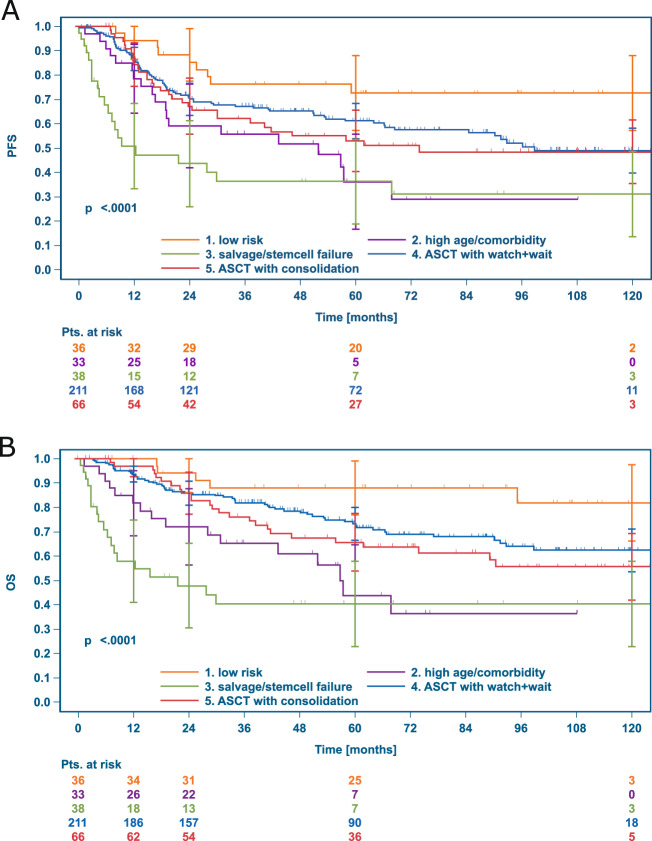
PFS and OS according to disease and treatment characteristics. **A** progression-free survival (PFS), **B** overall survival (OS), ASCT high-dose chemotherapy and autologous stem-cell transplantation, rrHL relapsed or refractory Hodgkin lymphoma.

With a median follow-up of 71.8 months for OS after first rrHL, 126 patients (30.8%) died, and 170 patients (41.6%) had a further PFS event after first rrHL. The Kaplan–Meier estimates for PFS and OS 12, 24, 60 and 120 months after first rrHL are reported for the total sample and the 5 previously defined patient subgroups of interest in Table 3. The 10-year PFS rate with a median follow-up of 70.6 months in the total sample was 48.2% (95% CI 41.9–54.2%, Fig. 2A) and 10-year OS 59.4% (95% CI 53.0–65.2%, Fig. 2B). Significantly different PFS and OS was observed in the previously defined five subgroups as depicted in Fig. 3, with most favorable outcomes observed in low-risk patients and those receiving ASCT without consolidation. Similarly, we found significant differences in PFS and OS among patients not receiving ASCT (Supplementary Fig. 1). Treatment-related mortality (TRM) was low and observed in nine patients (2%), mostly with intended ASCT (*n* = 8).

**Table 3:** PFS and OS up to 10 years after first rrHL.

	Group 1	Group 2	Group 3	Group 4	Group 5	Groups 3-5	Total
	low risk	high age/comorbidty	salvage/stem cell failure	ASCT with watch & wait	ASCT with RT consolidation	ASCT intended	
	*N* = 36	*N* = 33	*N* = 38	*N* = 211	*N* = 66	*N* = 315	*N* = 409
Progression-free survival							
12 months	94.3% (79.0–98.5%)	78.6% (60.2–89.2%)	50.8% (32.4–66.6%)	86.7% (81.2–90.7%)	84.4% (72.9–91.3%)	82.3% (77.5–86.2%)	82.7% (78.6–86.1%)
24 months	88.4% (72.0–95.5%)	59.0% (40.0–73.8%)	43.6% (25.8–60.1%)	70.0% (63.0–76.0%)	67.1% (54.1–77.2%)	66.5% (60.7–71.6%)	67.7% (62.6–72.2%)
60 months	72.6% (53.7–84.8%)	36.2% (17.7–55.0%)	36.3% (19.7–53.2%)	61.1% (53.4–68.0%)	53.0% (39.7–64.6%)	56.6% (50.3–62.3%)	56.5% (51.0–61.6%)
120 months	72.6% (53.7–84.8%)	n.a.	31.3% (15.0–48.7%)	49.0% (39.6–57.7%)	48.5% (35.0–66.6%)	47.2% (40.1–53.9%)	48.2% (41.9–54.2%)
Overall survival							
12 months	100.0% (100.0–100.0%)	81.7% (63.7–91.3%)	57.9% (39.3–72.7%)	93.6% (89.3–96.3%)	96.9% (88.1–99.2%)	90.5% (86.6–93.3%)	90.6% (87.3–93.1%)
24 months	94.1% (78.5–98.5%)	72.0% (53.0–84.4%)	47.8% (29.8–63.8%)	85.9% (80.2–90.0%)	85.9% (74.6–92.4%)	81.7% (76.8–85.7%)	82.4% (78.2–85.9%)
60 months	88.0% (71.2–95.3%)	43.8% (22.8–63.0%)	40.5% (23.2–57.1%)	73.3% (65.8–79.3%)	65.7% (52.3–76.2%)	68.0% (62.0–73.3%)	68.9% (63.6–73.6%)
120 months	81.8% (59.4–92.5%)	n.a.	40.5% (23.2–57.1%)	62.3% (53.0–70.3%)	55.6% (41.0–68.0%)	58.4% (51.2–64.9%)	59.4% (53.0–65.2%)

Kaplan–Meier estimates (95% confidence intervals).

*ASCT* high-dose chemotherapy and autologous stem-cell transplantation, *PFS* progression-free survival, *OS* overall survival, *95% CI* confidence interval, *n.a.* not applicable.

**Fig. 4:**
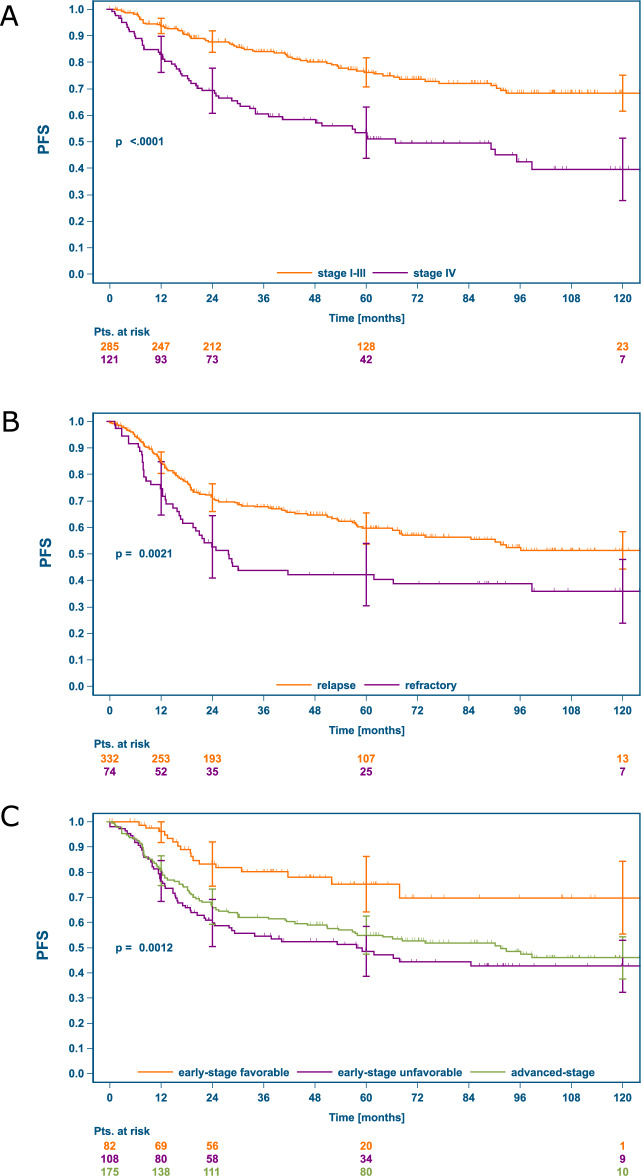
PFS after first rrHL according to the RFs stage IV, refractory disease and GHSG risk group at 1st-diagnosis. **A** stage IV vs. stage I-III at rrHL, **B**: refractory disease vs. TTR > 3 months, **C** PFS at rrHL according to risk-group at 1st diagnosis. PFS progression-free survival, rrHL relapsed or refractory Hodgkin lymphoma, RFs risk factors.

Significantly inferior outcomes were observed in patients with stage IV at rrHL and primary refractory patients experiencing relapse within three months after end of 1st-line treatment (i.e. TTR < 3 months). Inferior PFS (Fig. 4) and OS (Supplementary Fig. 2) in presence of the respective RFs was found in the total sample as well as the different cohorts. With intention to treat with ASCT, PFS in patients with stage IV disease appeared improved but remained statistically inferior compared to stage I-III (Supplementary Fig. 3). In patients with TTR ≤ 3 months, the outcome differences with intent to treat with or without ASCT seemed even more pronounced compared to patients with TTR > 3 months (Supplementary Fig. 3). Similarly, a significant influence of GHSG risk group at 1st-diagnosis on PFS (Fig. 4) and OS (Supplementary Fig. 2) after first rrHL was observed, with more favorable outcomes after initial early-stage favorable disease. In patients with the less completely documented risk factors bulk ≥5 cm and ECOG > 0 we observed no clear outcome differences according to intended treatment at rrHL (Supplementary Fig. 4).

## Discussion

The treatment landscape of 1st-line HL continues to change with therapeutic strategies and intensity increasingly tailored to the individual patients’ risk. With the approval of targeted agents, a similar trend is observed in rrHL. Characteristics and treatment patterns with associated outcomes are insufficiently studied for rrHL after contemporary 1st-line treatment. By thorough analysis of 409 patients with rrHL identified in 5,836 GHSG patients, the present study provides a robust benchmark of relapse characteristics, treatment patterns and outcomes after contemporary 1st-line therapy in a multicenter international setting. Patients with a broad age range of 18–77 years and heterogeneous presence of RFs such as bulk ≥5 cm (42%), stage IV (30%), extranodal disease (29%) or TTR ≤ 3 months (18%) were identified. Overall, the 10-year PFS and OS rates were 48.2% and 59.4%, respectively, highlighting the curative potential with varying 2nd-line HL treatments in a heterogenous patient population.

Salvage chemotherapy and ASCT are considered SOC in eligible rrHL patients based on superior PFS compared to salvage chemotherapy without ASCT [5, 6]. Much of the research in rrHL hence focused on the ASCT-eligible subgroup to optimize outcomes with this intensive treatment. The introduction of innovative salvage therapies aiming to achieve an optimal response prior to ASCT e.g., by incorporation of novel drugs is increasingly used to provide a risk-adapted treatment potentially mitigation the inferior outcomes in high-risk patients [28–31]. By utilizing targeted agents such as BV, anti-PD1 antibodies or others either alone, in combination with each other or together with conventional therapies such as bendamustine, ongoing studies aim to mitigate the inferior prognosis conferred by persistent PET-positivity in rrHL. High-risk features are currently additionally addressed by consolidative therapy such as a tandem-ASCT [32], consolidative RT peri-ASCT [33] and consolidative treatment with BV [21]. To guide these risk-adapted interventions, an international research group developed a simple, validated prognostic score for PFS and OS after ASCT based on five clinical RFs [15].

In the present study, patients successfully undergoing ASCT and not receiving consolidation due to lack of high-risk features conferred a rather favorable prognosis with 5-year PFS and OS of 61.1% and 73.3%, respectively. While survival rates of patients receiving consolidative RT peri-ASCT due to residual disease were similar at 24 months, these patients had inferior five-year survival rates (PFS: 53.0%, OS 65.7%), potentially hinting at a rather short-lived effect of localized consolidative treatment. While many studies in rrHL only report patients successfully undergoing ASCT, we specifically evaluated all patients in whom ASCT was intended to provide an “intention-to-treat” benchmark. In this large group of patients (*N* = 315), 38 patients could not undergo ASCT at first rrHL due to failure of salvage therapy/stem-cell mobilization and had poor survival outcomes (five-year PFS 36.3%, OS 40.5%). Patients successfully undergoing ASCT without the need for consolidation (*N* = 211, five-year PFS 61.1%, OS 73.3%) or with RT consolidation (*N* = 66, five-year PFS 53.0%, OS 65.7%) had more favorable outcomes. As expected, patients not undergoing ASCT due to age/comorbidities also experienced unfavorable outcomes (*N* = 33, five-year PFS 36.2%, OS 43.8%).

These findings are largely in line with previous studies, reporting worse outcomes in frail patients or with insufficient response to salvage treatment [15]. Our predefined subgroup analyses of patients with adverse RF confirmed the inferior outcomes in case of refractory HL, defined as TTR ≤ 3 months, or stage IV at relapse. Administration of ASCT in these subsets appears able to mitigate the inferior outcomes at least partially. We additionally observed inferior PFS and OS after rrHL in patients initially presenting with early-stage unfavorable or advanced-stage disease in contrast to patients with relapse after early-stage favorable HL. This observation corresponds well with a recent analysis, highlighting the favorable outcome in the latter population with non-inferiority of conventional chemotherapy vs. ASCT in terms of PFS [8]. Interestingly, and in line with other prior studies [8, 34], favorable survival rates were found in patients not undergoing ASCT due to low-risk disease with five-year PFS and OS of 72.6% and 88.0%, respectively.

The treatment landscape of rrHL is constantly changing and our cohort did only include few patients receiving BV consolidation after ASCT. In the pivotal international AETHERA trial, five-year PFS with BV consolidation was 59%, suggesting a substantial role in mitigating high-risk disease [35]. The increasing use of targeted agents also in the 1st-line setting e.g., by incorporating BV or anti-PD1 antibodies––although the latter to date off-label and mainly in the context of clinical research––on one hand might decrease the number of high-risk rrHL cases [36–38]. On the other hand the increasing proportion of patients already exposed to these agents poses to date largely unaddressed issues with regard to feasibility and efficacy of re-treatment in case of rrHL.

Our study builds upon a large international cohort of HL patients with detailed and prospective documentation of the disease course within the GHSG database with analysis of all evaluable first episodes of rrHL across risk groups. Inherent limitations of retrospective analyses such as partially missing data, insufficient documentation of treatment-associated toxicities except TRM, however, also apply to the present study. Such limitations also apply to the analysis of potential additional RFs such as anemia, which were not captured in a sufficient number of patients and hence could not be analyzed. The local assessment of response to rrHL treatment, with the vast majority of patients presumably evaluated by conventional CT instead of PET/CT and heterogeneous response criteria applied, limits further analysis and conclusions regarding this outcome measure. Additional potential limitations towards the generalizability of our data arise from the fact that all HD13-HD15 patients were eligible for clinical trial enrollment at 1st diagnosis and hence not necessarily reflect the general, completely unselected population of all patients with first rrHL. Patients with severe comorbidities or older patients beyond 75 years of age are likely underrepresented in the present analysis despite posing an ongoing clinical challenge. With consideration of these potential limitations, we are able to present long-term follow-up after rrHL arising after contemporary 1st-line treatments still used today with robust estimates of 10-year PFS and OS. More recent changes in the therapeutic landscape of rrHL are however underrepresented in our sample and the data hence primarily reflects outcomes with conventional therapies.

In conclusion, the present study reports comprehensive data on treatment patterns and outcomes including 10-year survival rates in distinct subgroups of rrHL patients after contemporary 1st-line treatment. Our analysis of 409 patients with rrHL highlights the curative potential of 2nd-line HL treatment, even without ASCT, with five-year PFS and OS of 56.5% and 68.9% observed in the total study population, respectively. In subset analyses we confirm previously identified RFs such as refractory disease or stage IV at relapse, with the former at least partially mitigated if intensified treatment with ASCT is intended. Patients successfully undergoing ASCT without the need for consolidation carry a favorable prognosis (five-year PFS 61.1%, five-year OS 73.3%), while outcomes in patients not receiving ASCT due to high age/comorbidities (five-year PFS 36.2%, five-year OS 43.8%) or failure of salvage therapy/stem-cell mobilization (five-year PFS 36.3%, five-year OS 40.5%) are inferior. Our study thereby underscores the unmet medical need in older or frail and refractory rrHL patients. In contrast, evaluation of deescalated therapies may be justified in more favorable risk groups such as rrHL after early-stage favorable HL, late relapse or with limited stage disease [39]. Finally, our data may serve as a benchmark analysis for evaluation of the relative efficacy of novel agents or therapeutic strategies in rrHL.

## Supplementary information

Supplementary

## Data Availability

The datasets generated and analyzed during the current study and single patient data can be made available upon reasonable request. Decisions regarding data sharing will be made on a case-by-case basis by the corresponding author considering data protection and other applicable regulations. Proposals may be submitted to the corresponding author.
