# Supplementary material for: Clinical outcomes of relapsed and refractory Hodgkin lymphoma patients after contemporary first-line treatment: a German Hodgkin Study Group analysis
Source: Leukemia. 2021 Oct 9;36(3):772–80. doi: 10.1038/s41375-021-01442-8 (PMC8885415; doi:10.1038/s41375-021-01442-8)
Supplement: Supplementary file 1 — Supplementary [file 41375_2021_1442_MOESM1_ESM.docx]

**Supplementary Material**

**Supplementary Figures**

**Supplementary Figure 1: PFS and OS according to reason for not receiving ASCT at first rrHL**

Panel A: progression free survival PFS, Panel B: overall survival OS, both from first rrHL

**Supplementary Figure 2: OS after first rrHL according to the RFs stage IV, refractory disease and risk group at 1^st^-diagnosis**

Panel A: stage IV vs. stage I-III at rrHL, Panel B: refractory disease vs. TTR > 3 months, Panel C: OS at rrHL according to risk-group at 1^st^ diagnosis. OS: overall survival, rrHL: relapsed or refractory Hodgkin lymphoma, RFs: risk factors

**Supplementary Figure 3: PFS in patients intended to treat with or without ASCT in the presence of risk factors stage IV disease at relapse and refractory disease (TTR ≥3 months)**

Panel A: PFS according to stage in rrHL patients not intended to treat with ASCT, Panel B: PFS according to stage in rrHL patients intended to treat with ASCT, Panel C: PFS according to TTR in rrHL patients not intended to treat with ASCT, Panel D: PFS according to TTR in rrHL patients intended to treat with ASCT. PFS: progression-free survival from first rrHL, ASCT: high-dose chemotherapy and autologous stem-cell transplantation, rrHL: relapsed or refractory Hodgkin, TTR: time to relapse, refractory disease defined as TTR ≤3 months after end of 1^st^-line treatment.

**Supplementary Figure 4: PFS in patients intended to treat with or without ASCT in the presence of risk factors at relapse.**

Panel A: PFS according to bulk ≥ 5cm in rrHL patients not intended to treat with ASCT, Panel B: PFS according to bulk ≥ 5cm in rrHL patients intended to treat with ASCT, Panel C: PFS according to ECOG > 0 in rrHL patients not intended to treat with ASCT, Panel D: PFS according to ECOG in rrHL patients intended to treat with ASCT, E: PFS according to B symptoms in rrHL patients not intended to treat with ASCT. F: PFS according to B symptoms in rrHL patients intended to treat with ASCT. PFS: progression-free survival from first rrHL, ASCT: high-dose chemotherapy and autologous stem-cell transplantation, rrHL: relapsed or refractory Hodgkin lymphoma

**Supplementary Tables**

**Supplementary Table 1: Characteristics of the five major patient subgroups**

|  | | | | | | |
| --- | --- | --- | --- | --- | --- | --- |
|  | low risk (N=36) | high age/ comorbidities (N=33) | salvage/stem-cell failure (N=38) | ASCT + w&w (N=211) | ASCT + RT consolidation (N=66) | Total (N=384) |
| **Sex** |  |  |  |  |  |  |
| female | 15 (42%) | 14 (42%) | 13 (34%) | 69 (33%) | 25 (38%) | 136 (35%) |
| male | 21 (58%) | 19 (58%) | 25 (66%) | 142 (67%) | 41 (62%) | 248 (65%) |
|  |  |  |  |  |  |  |
| **Age at rrHL** |  |  |  |  |  |  |
| Mean (SD) | 36.8 (11.7) | 61.6 (10.8) | 37.7 (12.9) | 37.1 (12.0) | 34.0 (11.1) | 38.7 (13.7) |
| Median | 35.1 | 63.7 | 38.0 | 36.7 | 31.9 | 37.6 |
| Range | (18.9-56.5) | (29.8-75.2) | (18.4-58.7) | (18.6-66.0) | (20.3-66.3) | (18.4-75.2) |
|  |  |  |  |  |  |  |
| **Age >60 years at rrHL** |  |  |  |  |  |  |
| No | 36 (100%) | 12 (36%) | 38 (100%) | 206 (98%) | 65 (98%) | 357 (93%) |
| Yes | 0 (0%) | 21 (64%) | 0 (0%) | 5 (2%) | 1 (2%) | 27 (7%) |
|  |  |  |  |  |  |  |
| **Clinical stage at 1st diagnosis** |  |  |  |  |  |  |
| missing | 0 | 0 | 0 | 1 | 1 | 2 |
| I | 7 (19%) | 9 (27%) | 0 (0%) | 8 (4%) | 2 (3%) | 26 (7%) |
| II | 12 (33%) | 14 (42%) | 15 (39%) | 114 (54%) | 30 (46%) | 185 (48%) |
| III | 10 (28%) | 6 (18%) | 9 (24%) | 44 (21%) | 12 (18%) | 81 (21%) |
| IV | 7 (19%) | 4 (12%) | 14 (37%) | 44 (21%) | 21 (32%) | 90 (24%) |
|  |  |  |  |  |  |  |
| **B symptoms**  **at 1st diagnosis** |  |  |  |  |  |  |
| missing | 0 | 0 | 0 | 1 | 1 | 2 |
| no | 28 (78%) | 24 (73%) | 17 (45%) | 105 (50%) | 23 (35%) | 197 (52%) |
| yes | 8 (22%) | 9 (27%) | 21 (55%) | 105 (50%) | 42 (65%) | 185 (48%) |
|  |  |  |  |  |  |  |
| **Time to relapse** |  |  |  |  |  |  |
| Missing | 0 | 1 | 0 | 2 | 0 | 3 |
| ≤ 3 months | 4 (11%) | 2 (6%) | 12 (32%) | 32 (15%) | 19 (29%) | 69 (18%) |
| 3-12 months | 17 (47%) | 3 (9%) | 8 (21%) | 68 (33%) | 24 (36%) | 120 (31%) |
| >12 months | 15 (42%) | 27 (84%) | 18 (47%) | 109 (52%) | 23 (35%) | 192 (50%) |
|  |  |  |  |  |  |  |
| **Clinical stage at rrHL** |  |  |  |  |  |  |
| missing | 0 | 0 | 1 | 0 | 2 | 3 |
| I | 13 (36%) | 9 (27%) | 4 (11%) | 31 (15%) | 7 (11%) | 64 (17%) |
| II | 12 (33%) | 8 (24%) | 13 (35%) | 59 (28%) | 24 (38%) | 116 (30%) |
| III | 5 (14%) | 8 (24%) | 8 (22%) | 51 (24%) | 13 (20%) | 85 (22%) |
| IV | 6 (17%) | 8 (24%) | 12 (32%) | 70 (33%) | 20 (31%) | 116 (30%) |
|  |  |  |  |  |  |  |
| **B symptoms at rrHL** |  |  |  |  |  |  |
| Missing | 14 | 16 | 10 | 69 | 19 | 128 |
| No | 13 (59%) | 10 (59%) | 14 (50%) | 109 (77%) | 27 (57%) | 173 (68%) |
| Yes | 9 (41%) | 7 (41%) | 14 (50%) | 33 (23%) | 20 (43%) | 83 (32%) |
|  |  |  |  |  |  |  |
| **Bulk ≥ 5cm at rrHL** |  |  |  |  |  |  |
| missing | 14 | 18 | 17 | 97 | 25 | 171 |
| No | 11 (50%) | 11 (73%) | 7 (33%) | 76 (67%) | 20 (49%) | 125 (59%) |
| Yes | 11 (50%) | 4 (27%) | 14 (67%) | 38 (33%) | 21 (51%) | 88 (41%) |
|  |  |  |  |  |  |  |
| **EN disease at rrHL** |  |  |  |  |  |  |
| Missing | 14 | 11 | 16 | 66 | 20 | 127 |
| No | 18 (82%) | 13 (59%) | 11 (50%) | 106 (73%) | 34 (74%) | 182 (71%) |
| Yes | 4 (18%) | 9 (41%) | 11 (50%) | 39 (27%) | 12 (26%) | 75 (29%) |
|  |  |  |  |  |  |  |
| **ECOG at rrHL** |  |  |  |  |  |  |
| Missing | 22 | 18 | 17 | 91 | 39 | 187 |
| 0 | 12 (86%) | 11 (73%) | 15 (71%) | 94 (78%) | 21 (78%) | 153 (78%) |
| 1 | 0 (0%) | 1 (7%) | 2 (10%) | 14 (12%) | 4 (15%) | 21 (11%) |
| 2 | 2 (14%) | 3 (20%) | 3 (14%) | 12 (10%) | 2 (7%) | 22 (11%) |
| 3 | 0 (0%) | 0 (0%) | 1 (5%) | 0 (0%) | 0 (0%) | 1 (1%) |

Abbreviations ASCT: high-dose chemotherapy and autologous stem-cell transplantation; rrHL: relapsed or refractory classical Hodgkin lymphoma; EN: extranodal disease; SD: standard deviation

**Supplementary Table 2: Treatment administered for first rrHL if ASCT was not intended**

|  | low risk (N=36) | high age/ comorbidities (N=33) |
| --- | --- | --- |
| Polychemotherapy | 15 (42%) | 17 (52%) |
| Targeted agent | 0 (0%) | 2 (6%) |
| Radiotherapy (RT) | 18 (50%) | 4 (12%) |
| Palliative single agent chemotherapy/steroids | 1 (3%) | 4 (12%) |
| Polychemotherapy + RT | 2 (6%) | 6 (18%) |
|  |  |  |

**Supplementary Table 3: Reasons for not undergoing ASCT**

| reason | N=111 |
| --- | --- |
| insufficient response to salvage chemotherapy | 30 (27%) |
| age | 24 (22%) |
| only RT at rrHL due to localized relapse | 21 (19%) |
| patients wish | 18 (16%) |
| comorbidities | 10 (9%) |
| late relapse (TTR >5 years) | 10 (9%) |
| insufficient apheresis (i.e. stem-cell failure) | 3 (3%) |
| other/unknown | 11 (10%) |

Abbreviations: RT: radiotherapy, TTR: time to relapse after end of 1^st^-line treatment; multiple reasons possible; only patients initially treated in the HD13-15 trials
